# Evaluation of a group-based online informed consent conversation (eConsent) in participants from a low-risk vaccination clinical trial

**DOI:** 10.1186/s13063-024-08367-4

**Published:** 2024-08-07

**Authors:** Ngoc H. Tan, Melvin Lafeber, Roos S. G. Sablerolles, Isabelle Veerman Roders, Anna van de Hoef, Karenin van Grafhorst, Leo G. Visser, Douwe F. Postma, Abraham Goorhuis, Wim J. R. Rietdijk, P. Hugo M. van der Kuy

**Affiliations:** 1 Department of Hospital Pharmacy, Erasmus Medical Center, Rotterdam, the Netherlands; 2 Department of Internal Medicine, Erasmus Medical Center, Rotterdam, the Netherlands; 3 Department of Infectious Diseases, Leiden University Medical Center, Leiden, the Netherlands; 4 Department of Internal Medicine and Infectious Diseases, University Medical Center Groningen, Groningen, the Netherlands; 5 Center of Tropical Medicine and Travel Medicine, Department of Infectious Diseases, Amsterdam University Medical Centers, Amsterdam, the Netherlands; 6 Infection & Immunity, Amsterdam Public Health, University of Amsterdam, Amsterdam, the Netherlands

**Keywords:** Group, eConsent, Informed consent

## Abstract

**Background:**

Electronic informed consent (eConsent) usage has expanded in recent years in Europe, especially during the pandemic. Slow recruitment rate and limitations in participant outreach are the challenges often faced in clinical research. Given the benefits of eConsent and group counselling reported in the literature, group eConsent was implemented in recruitment for the SWITCH-ON study. We aim to explore the experience of participants who attended group eConsent for the SWITCH-ON study and evaluate its potential for future use.

**Methods:**

SWITCH-ON study aims to analyse the immunogenicity of a healthy population following bivalent COVID-19 booster vaccination. Four hundred thirty-four healthcare workers aged 18–65 were successfully recruited and sent a questionnaire about their experience with group eConsent. Out of 399 completed questionnaires (response rate 92%), 39 participants did not join group eConsent. The remaining 360 responses were included in the final analysis. Quantitative and qualitative data were reported using descriptive statistical analysis and thematic analysis respectively.

**Results:**

Participants found that group eConsent was an efficient method that it allowed them to hear each other’s questions and concerns and created a sense of togetherness. However, limited privacy, barriers to asking questions in a group, and peer pressure can limit the use of group eConsent. One hundred sixty-five (46%) participants thought that group eConsent was suitable to recruit participants with diseases or conditions, while 87 (24%) reported limitations with this method. The remaining participants suggested that applicability of group eConsent depended on the diseases or conditions of the study population, and one-to-one conversation should always be available. Participants who had experienced both one-to-one and group eConsent shared different preferred consent formats for future studies.

**Conclusion:**

Group eConsent was positively evaluated by the participants of a low-risk vaccination study. Participants advised using webinars to provide general information about the study, followed by an individual session for each participant, would retain the benefits of group eConsent and minimise the limitations it posed. This proposed setting addresses privacy questions and makes group eConsent easier to implement.

**Trial registration:**

ClinicalTrials.gov NCT05471440 (registered on 22nd of July, 2022).

**Supplementary Information:**

The online version contains supplementary material available at 10.1186/s13063-024-08367-4.

## Introduction

Informed consent is one of the basic ethical principles in medical research as stated in the Declaration of Helsinki [1]. Depending on the scale of the study, the recruitment and consent process can be lengthy and cumbersome. In some studies, the extensive inclusion and exclusion criteria can also add to the recruitment duration. Furthermore, difficulty in recruiting participants, study funding limitations and lack of trained staff have been recognised as challenges for clinical studies [2, 3]. Participant-related recruitment challenges such as time required for the study and lack of knowledge about the research also contribute to failures of study recruitment [4]. Many research groups have diverged from the traditional paper-based informed consent process and digitalised their approach to shorten the recruitment period and enable more participants to join, thus reducing the incidence of study delay or termination due to lack of participants, and the costs involved with this [5].

The European Medicine Agency characterised electronic informed consent (eConsent) as a two-part process in a draft guidance [6]. The first part involves using digital methods such as video and (non-)interactive multimedia to educate the study participants and provide them with an appropriate level of understanding of the study. The second part is capturing an electronic signature (eSignature) after participants have had sufficient time and understanding to make an informed decision. In this article, the term “eConsent” is used to cover both parts of the process.

eConsent has been established in the United States of America (US) since the publication of a joint guidance by the Food and Drug Administration (FDA) and the Department of Health and Human Services (HHS) Office for Human Research Protections (OHRP) in 2016 [7]. As a result, the process has been harmonised across the US. On the other hand, variations were observed across the European Union (EU), with some countries accepting and regulating eConsent, some accepting without explicit regulation, or some not accepting eConsent [8]. The coronavirus disease 2019 (COVID-19) pandemic led to physical distancing or “lockdown” measures, which were introduced periodically across many countries, subsequently affecting the timelines of clinical studies. However, the pandemic was also the catalyst enabling the rapid expansion of eConsent use in the EU.

eConsent has been reported as a useful tool to improve research workflow, reduce administrative errors often reported with paper-based consent, reduce travel time, and provide greater reach to the wider population [9–13]. Although eConsent offers many practical benefits, its use has not been reported in a group setting. The use of a group setting has been reported to enhance patients’ knowledge of the subject, leading to more effective decision making in prenatal screenings and counselings [14–16]. For clinical studies, seminars or group recruitment strategies were useful in reducing workload for researchers [17–19]. In July 2022, the Central Committee on Research Involving Human Subjects (CCMO) published guidance for eConsent in the Netherlands [20]. It outlines a framework for the Medical Ethics Committee (METC) to review studies using eConsent as part of their recruitment process. The SWITCH-ON study started recruiting participants shortly after the introduction of this guideline, which allowed us to incorporate eConsent into our study [21]. The advantages of eConsent and group recruitment were favourable for study recruitment in a short time period which was why group eConsent setting was chosen as the recruitment method. Here, we explore the experience of participants with eConsent in a group setting and discuss its future use in research. We hypothesised that participants of our low-risk vaccination trial would positively evaluate the group eConsent procedure.

## Methods

### Study design and participants

In this study, participants from the SWITCH-ON study, who have signed the informed consent form, were invited to complete a digital questionnaire about their experience with group eConsent [21, 22]. SWITCH-ON is an open-label, multicentre, randomised controlled trial which adheres to the declaration of Helsinki. The aim of the study is to analyse immunogenicity of bivalent booster vaccination against COVID-19 in healthy healthcare workers (HCWs). The study was funded by the Netherlands Organization for Health Research and Development (ZonMw grant number 10430072110001). Before the start of the recruitment, as a healthcare institute, we were advised that HCWs would be recommended to receive further booster in autumn of 2022 as part of the national vaccination campaign. Therefore, 400 participants had to be recruited before the vaccination campaign started in autumn 2022. In other words, study recruitment for SWITCH-ON had to be completed within 4 weeks (September to October 2022).

The recruitment and informed consent process in the SWITCH-ON study is briefly described here and the full details of the study protocol can be found in our previous publication [21]. HCW aged between 18 and 65 years from four academic hospitals in the Netherlands were invited to join the SWITCH-ON study. Four hundred participants were required for the study to achieve its power calculation target. After enrolment, participants would be randomised equally to two groups: direct boost group (booster vaccination in October 2022) and postponed boost group (booster vaccination in December 2022). If eligible, healthcare workers were sent the participant information leaflet and invited to join a group eConsent session via an online platform (Microsoft Teams). The invitation provided participants information about possible dates and times for the informed consent session and specified that the session would be joined by other participants. Participants were also offered one-to-one (1:1) conversation (online or face-to-face) as an alternative arrangement if that was preferred.

The eConsent sessions were initially set up with a maximum of 10 participants per session. However, due to the recruitment time constraint (4 weeks), two sessions were adjusted to allow up to 15 participants to join. Participants were asked to turn their camera on while attending the session so that their identity could be verified. During the session, one of the investigators presented all the essential information about the study and answered questions from participants. At the end of the session, participants were offered three options: (1) remaining in the online session if they would like to sign the consent form, (2) leaving the session if they do not want to participate, or (3) leaving the session if they need more time to reflect or raise additional questions. For participants who chose option 1, they were advised to stay in the session until the signed consent form was received and checked by the research team. Afterwards, participants could leave the session. Participants who chose option 2 and 3 were advised to send an email to the research team about their decision. Figure S1 (Supplementary) summarises the study recruitment process.

After randomisation, participants would receive multiple questionnaires for baseline characteristics, personal data, COVID-19 infection status and appointment invitations. Due to the number of questionnaires participants need to fill in and the tight schedule for the study visits in the first 28 days, participants were only sent the questionnaire about their experience with group eConsent between 1 and 2 months after attending their eConsent session. A reminder was sent to participants if no response was received within 2 weeks since the first eConsent questionnaire email. Participants were assumed to decline participation in this survey if they did not respond 2 weeks after the reminder was sent. Figure S2 (Supplementary) shows the SWITCH-ON study schedule up to January 2023.

### Data analysis

The questionnaire consisted of multiple choice, numeric rating scale (NRS) and open-ended questions to grasp the experience of participants with group eConsent. The details of the questionnaire can be found in Supplementary Appendix 1. Descriptive analysis was used to report the baseline characteristics and quantitative data. For continuous baseline variables and the scores of the NRS (0 to 10, negative to positive), mean and standard deviation (SD) were reported if they had a normal distribution. Otherwise, median and interquartile range (IQR) were reported. For categorical baseline variables, they were reported as count with percentage. The answers in the multiple-choice questions were reported as count and percentage. Thematic analysis was used for qualitative data of the open-ended questions. All quotations for qualitative data are translation of participants’ response from Dutch by author N.H.T and A.V.D.H.

## Results

**Fig. 1:**
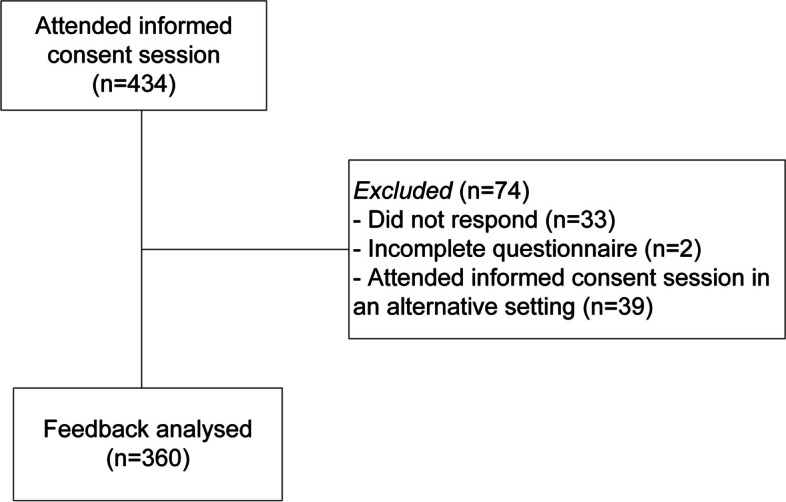
Flowchart of study inclusion for the final analysis

After the initial screening, 434 participants enrolled into the study out of 519 eligible participants (consent percentage 83.6%). Out of the 434 questionnaires sent, 401 were returned. However, 2 questionnaires were not answered completely and were excluded. Therefore, 399 completed questionnaires were received. Thirty-nine (9.8%) participants attended the informed consent session in an alternative setting, e.g. 1:1 phone conversation, in-person 1:1 meeting. Response from 360 (90.2%) participants were included in the final analysis (Fig. 1).

In Table 1, we presented the baseline characteristics of the 360 participants. The number of female participants is higher than male participants, which is the same for the SWITCH-ON study population. This is the reflection of biological sex of the healthcare professional proportion of the Netherlands. More than half of participants recalled being in a group of 5 to 10. About 3.3% of participants reported being in a group with more than 15 participants. One hundred ninety-four (53.9%) participants attended the session from home, while 163 (45.3%) participants attended the session at their workplace.

**Table 1:** Baseline information of participants and general information about the group session

*Participant characteristics*	**Group eConsent** (*n* = 360)
***Sex***	
Male	90 (25.0%)
Female	270 (75.0%)
***Age (in years)***	47 [37–53]^a^
***Occupation***	
Administrator/assistant	55 (15%)
Doctors	22 (6%)
Facility workers	9 (3%)
Managers/team leaders	41 (11%)
Nurses	27 (8%)
Support staff in clinics/urgent care unit	5 (1%)
Support staff in outpatient clinics	4 (1%)
Researchers	78 (22%)
Others^b^	117 (33%)
Unknown	2 (1%)
***Number of participants per digital session***	
Less than 5 participants	101 (28.1%)
Between 5 to 10 participants	204 (56.7%)
Between 11 to 15 participants	44 (12.2%)
More than 15 participants	11 (3.1%)
***Location where participant joined the digital session***	
Home	194 (53.9%)
Work	163 (45.3%)
Other	3 (0.8%)

^a^Age reported here as median [IQR]

^b^Others include occupations such as data manager, research analyst, communication advisor, receptionist, electrical engineer, and legal advisor

### Overview of group eConsent

When participants were asked about their experience with having others in their consent session on a scale of 0 to 10 (negative to positive), the overall score was 8 [IQR: 6 to 9]. This showed that the experience leaned towards positive. The factors influencing patients’ score were explored further by examining the advantages and disadvantages participants experienced in group eConsent.

### Advantages

#### Input from others

This dominant theme was recorded from participants’ response: “It is indeed nice to hear others’ questions. In this way, more questions can be asked and answered, which ultimately makes me better informed”. Furthermore, participants also found that some questions asked by others were useful and were not something they had thought about. Some participants found that after gaining more information through listening to others questions and answers, they could ask more questions to deepen their understanding of the study.

#### Efficient and flexible

Many participants found that group eConsent gave them more flexibility in location choice and reduced travel time, thus making it more efficient: “*Flexible time and location- Online possibility*”. Additionally, time saving was reported from reducing travel time and the joint explanation and efficient question and answer format of the group settings.

#### Sense of togetherness

This was described positively by some participants: “It was quite nice to see other fellow participants and it was also nice that together we can make this study possible”. The same feeling was also described as being part of the team supporting the research.

### Disadvantages

#### Privacy

Unlike the traditional 1:1 informed consent, completing the session as a group touched on the question of privacy, which was outlined by many participants: “For certain studies I would indeed not want others to see that I am participating. For this study, I don’t find that a problem, but when it comes to studies in a certain patient population, for example, I wouldn’t appreciate potentially running into someone I know”. Moreover, participants of the SWITCH-ON study were staff of four academic hospitals, which posed the possibility of participants recognising current or former colleagues.

#### Barriers to asking questions

Being in a group can be a barrier for participants to ask questions, especially ones that may only be relevant to themselves, e.g. logistics questions. This in turn increased the threshold of how comfortable one felt to ask questions in a group: “I had a logistics question that was only relevant to me, so I felt like I was weighing down other participants by talking about my logistics question in detail”.

#### Session length

Although the information given in all informed consent sessions was the same, participants received and understood the information often at different paces and depths. Some have already read the participant information leaflet and had no further questions, while others preferred the information to be rephrased differently for better understanding. Depending on which side of the spectrum participants were at, the informed consent session would be felt differently: “Questions that did not interest me or were not applicable were also asked. This was inevitable of course, but it made everything longer than it should have been for me”.

#### Peer pressure

Although the presence of other participants in the session was viewed mostly positively in the NRS, this should not diminish the potential negative effect on participants: “Peer pressure to give consent, not necessarily experienced that way myself, but I did notice that it is harder not to give consent”. Furthermore, the decision or attitude of others in the group towards the study can influence one’s decision: “Maybe it can be harder to be independent when you have to make a decision, or the decisions of others can influence yours”.

### Group eConsent in studies involving participants with a disease or condition

Participants in the SWITCH-ON study were healthy healthcare workers between the age of 18 and 65 years old. Due to these specific characteristics of the SWITCH-ON study population, we decided to explore the view of participants regarding the use of group eConsent in participants with a condition or disease. One hundred sixty-five (46%) participants thought that group eConsent was suitable to be used in recruitment involving participants with a condition or disease, while 87 (24%) participants disagreed. The remaining participants chose to provide more context about their choice, which were summarised in the following themes:

#### Study population

Participants found that the design of the consent session should be tailored to the study population: “I think that depends on what kind of disease is involved. For some diseases, people can be ashamed, so maybe they don’t like being recognised. […] For diseases that people are generally not ashamed of, I think it is generally a good possibility. Finally, I think you should also consider the age: diseases that occur mainly in elderly people are less suitable for Teams sessions because they may not be used to it and will experience technical problems. […]”.

#### Group eConsent as an option

Participants welcomed the use of group eConsent with the condition that personal conversations were still offered: “As long as there is always the possibility for 1:1 conversation”.

### Group eConsent in participants who have previously attended face-to-face session for other studies

Out of 360 participants, 182 (50.6%) have attended a face-to-face informed consent session for other studies. Those participants were asked a further question about their future preference for the design of the consent sessions. The percentages reported in the following section were calculated based on 182 participants, not the total 360 participants. The numbers of participants being interested in either online or face-to-face 1:1 conversation was similar, 24 (13.2%) and 25 (13.7%). Face-to-face consent with one or two other participants was chosen by 30 (16.5%) participants. Finally, 103 (56.6%) participants were open for eConsent with more than two people (group eConsent).

Some participants explained their choice by stating that group session could be used to provide background information and the purpose of the study, which should be followed by individual conversations for personal questions. Other participants stated that they were open for all informed consent settings.

### Study logistics

In addition to participants’ experience with group eConsent, logistics surrounding the group eConsent setup was also examined. Participants were informed about the group eConsent in the email invitation for the consent session. In this email, they were also advised about the option of 1:1 conversation. Most participants, 314 (87.2%), were aware of the 1:1 option but did not need it. The option was utilised by 8 (2.2%) participants. A total of 38 (10.48%) participants responded not being aware of this option, one of whom had wanted a private conversation.

After participants have asked all the questions in the session and decided that they would continue with the study, they had to remain in the session until the researchers received their consent form. The experience of having to stay on the online platform until signed consent form was received had an overall score of 8 (IQR: 5 to 10).

## Discussion

In this study, we found that group eConsent was positively evaluated by many participants for its advantages, but others also voiced their uncertainties towards this design. On one hand, participants found the group setting efficient, in that it is useful to hear others’ input and that it created a sense of togetherness. On the other hand, limited privacy, barriers to asking questions, and peer pressure can limit wider implementation of group eConsent.

Despite others’ input generally being considered an advantage, if questions asked were personal (such as logistics), some participants found them irrelevant and unnecessarily prolonging the session. The fear of taking up time with irrelevant questions can create a barrier to asking them in a group setting. This barrier might lead to a lack of information which could be important to participants and consequently affect their decision to participate in the study. On the contrary, in a 1:1 setting, participants could be more comfortable in asking specific questions relating to their own circumstance or condition, which enhanced their understanding and the decision-making process. However, group eConsent has enabled us to reach out to more participants and achieved the recruitment target within 4 weeks.

In a group environment, regardless of if it is in person or digital, participants may feel obliged to enrol in a study under peer pressure. It is important to create a secure environment for participants to make their own decision. In that regard, future group eConsent could start with a webinar, where participants can remain anonymous, followed by individual sessions for personal questions. During the webinar, participants can send in their questions, and if those are relevant to other participants, the researcher can answer them in the general session. In this way, participants still have the benefits of hearing questions from others and the comfort of asking follow-up questions in their individual session. This setting addresses the uncertainty about confidentiality, especially in study population with diseases or conditions. Furthermore, peer pressure would be minimised as participants will not know about the decision of others when they are in their own digital space. This design will match the responses from participants who previously attended 1:1 informed consent conversation for other studies. Those participants had the opportunity to reflect on their experience in both 1:1 and group eConsent. They expressed willingness to participate in future group eConsent sessions including both physical and digital settings. However, this would depend on the nature of the study and the availability of 1:1 conversation.

Clear communication is important during the recruitment period. Although participants were informed in the study invitation and at the beginning of each group eConsent session about the option of 1:1 conversation, participants could still miss out on important logistics information. Thirty-eight (10.48%) participants in this study reported not being aware of the 1:1 conversation option, and one of them would have chosen a private conversation. These cases indicated that more emphasis is needed to ensure participants are well-informed about their choices. Organisers for future group eConsent sessions should emphasise all possible arrangements (1:1 or group, physical or digital) at every stage leading to the informed consent session. We suggest all available options be mentioned again at the booking stage of informed consent appointment.

By optimising the process of group eConsent as described above, we will retain the benefits of group eConsent while addressing participants’ concerns recorded in the questionnaires, especially the question about confidentiality. In a larger legal context, guidelines for eConsent published by the CCMO have paved way for wider implementation of eConsent in the Netherlands. The guidelines should be expanded to cover group eConsent and provide clarity for researchers.

### Strengths, limitations, and future research

The questionnaire had a high response rate (92%), and therefore the results in this paper reflected the point of view of most participants in our study who attended the group eConsent. The applicability of group eConsent derived from this paper for other studies should take into account the limitations of the study population and study design. Firstly, participants from SWITCH-ON study were healthcare professionals between 18 and 65 years old. Licenced COVID-19 booster vaccinations used in the study were also recommended in the national vaccination campaign. These two factors may contribute to the high consent percentage of 83.6% and high consent rate, i.e. 434 participants gave consent per month (or 4 weeks). These are considerably higher than reported in a recent review in which the median consent percentage was 72% (IQR: 50–88%) and the median recruitment rate (participants per centre per month) was 0.95 (IQR: 0.42–2.60) [23]. If a study with a known low consent rate uses group eConsent for recruitment, the efficiency observed in the SWITCH-ON study may not be achieved. Secondly, healthy participants of the SWITCH-ON study do not face the same social stigma as in some diseases, which allowed them to be more open to the group setting. Thirdly, participants working in academic hospitals were exposed to regular use of computers, thus eConsent may not be applicable for research involving participants with lower technology literacy or impaired dexterity. Age and occupation are important factors to consider in assessing the level of technological acceptance. Lastly, the study was not designed to compare group eConsent with other forms of informed consent such as 1:1 in-person, 1:1 online, or in-person group meeting. We focused on exploring participants’ views and acceptance towards group eConsent as an informed consent method. Further research involving comparison between different informed consent methods will generate deeper insight of participants’ perception and acceptance towards group eConsent. Additionally, the cost of eConsent platform or eSignature subscription should be considered for the overall cost of the study recruitment.

## Conclusion

This study provided us insight into participants’ experience and perception towards group eConsent. We are accelerating into the age of digital health and embracing technological advances such as eConsent enables us to reach a wider population, improving the diversity of the participant cohort. When rapid development of studies is time-critical, group eConsent can aid researchers in achieving the recruitment target and reduce the risk of undue delay. Overall, group eConsent was viewed positively from participants’ perspective, which suggests that it can be an appropriate tool for future study recruitment. Wider implementation of this tool must consider the demographics of the study population and adhere to local research ethical framework and guidelines. A hybrid model consisting of in-person and digital environments should be considered to enhance participants’ experience and improve research workflow.

### Supplementary Information

Supplementary Material 1: Figure S1. eConsent process in the study. Figure S2. Study recruitment timeline. Appendix 1. Questionnaire in English.

## Data Availability

Deidentified individual participant data and other supporting documents will be made available upon requests made to the corresponding author.
