# Supplementary material for: Evaluation of a group-based online informed consent conversation (eConsent) in participants from a low-risk vaccination clinical trial
Source: Trials. 2024 Aug 7;25:528. doi: 10.1186/s13063-024-08367-4 (PMC11304818; doi:10.1186/s13063-024-08367-4)
Supplement: Supplementary file 1 — Supplementary Material 1: Figure S1. eConsent process in the study. Figure S2. Study recruitment timeline. Appendix 1. Questionnaire in English. [file 13063_2024_8367_MOESM1_ESM.pdf]

Evaluation of a group-based online informed consent conversation (eConsent) in participants from a vaccination clinical trial: a mixed method study

Ngoc H. Tan[1] MPharm., Melvin Lafeber[1,2] M.D., Ph.D., Roos S.G. Sablerolles[1] M.D, Isabelle Veerman Roders[1] BSc., Anna van de Hoef[1], Karenin van Grafhorst[1] BSc., Leo G. Visser[3] M.D., Ph.D., Douwe F. Postma[4] M.D., Ph.D., Abraham Goorhuis[5,6] M.D., Ph.D., Wim J.R. Rietdijk[1], Ph.D., P. Hugo M. van der Kuy[1], Pharm.D., Ph.D.

[1]Department of Hospital Pharmacy, Erasmus Medical Center, Rotterdam, the Netherlands

[2]Department of Internal Medicine, Erasmus Medical Center, Rotterdam, the Netherlands

[3]Department of Infectious Diseases, Leiden University Medical Center, Leiden, the Netherlands

[4]Department of Internal Medicine and Infectious Diseases, University Medical Center Groningen, Groningen, the Netherlands

[5]Center of Tropical Medicine and Travel Medicine, Department of Infectious Diseases, Amsterdam University Medical Centers, Amsterdam, the Netherlands

[6]Infection & Immunity, Amsterdam Public Health, University of Amsterdam, Amsterdam, the Netherlands

Corresponding author: P.H.M. van der Kuy, Department of Hospital Pharmacy, Erasmus Medical Center, Rotterdam, 3015 GD, the Netherlands. E-mail: h.vanderkuy@erasmusmc.nl

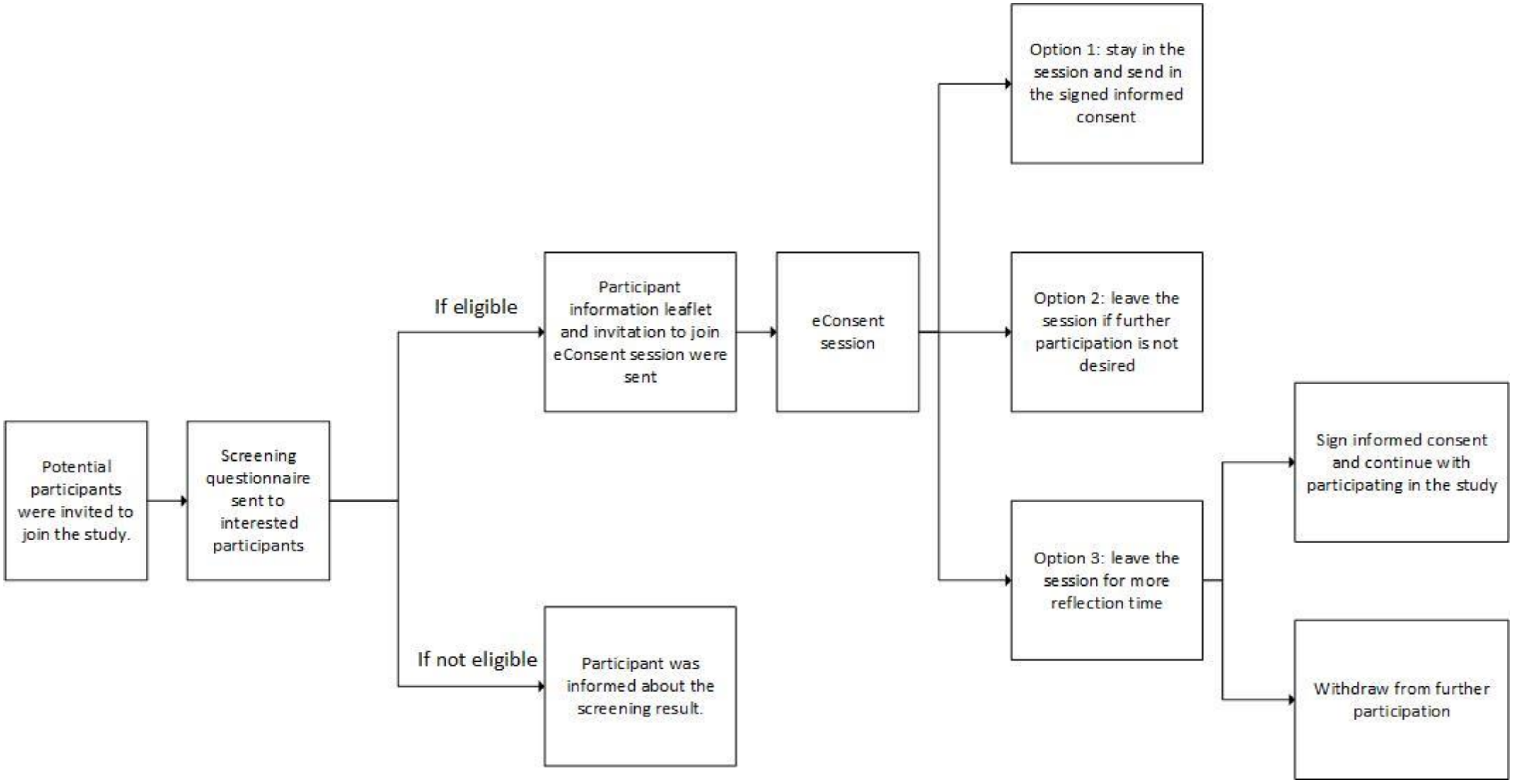

Figure S1. eConsent process in the study.

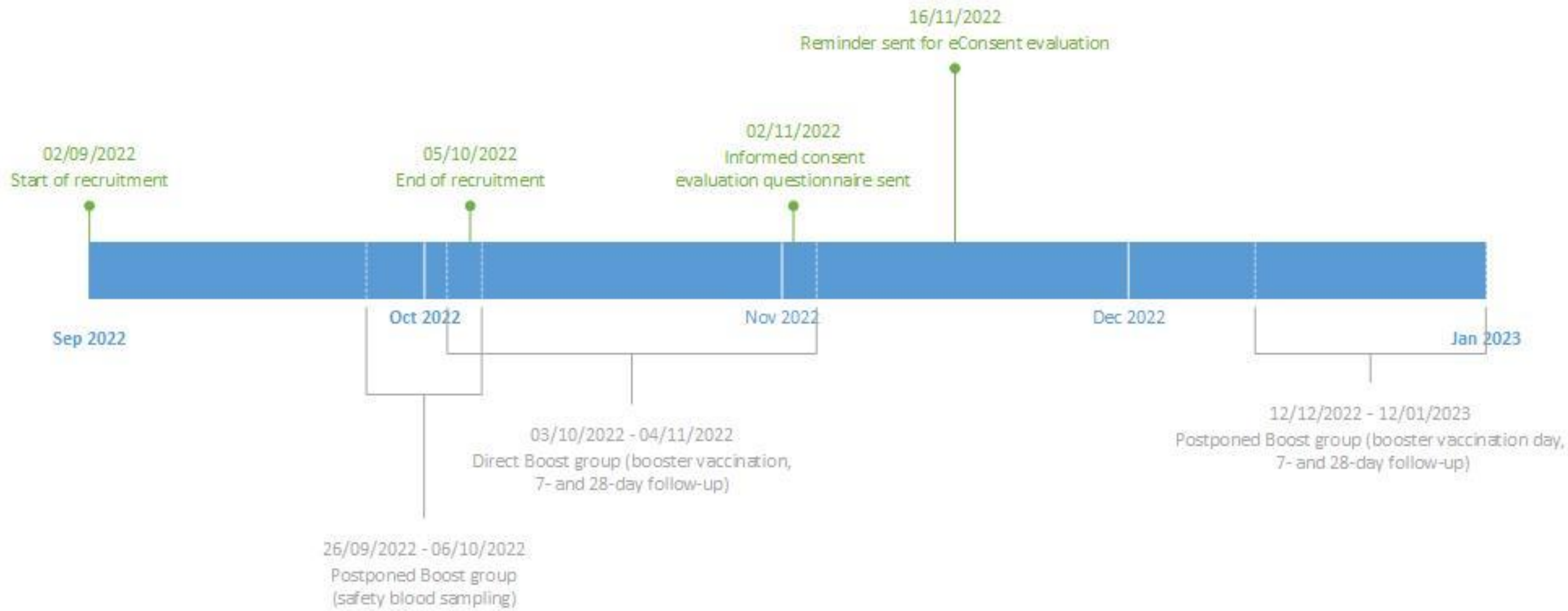

Figure S2. Study recruitment timeline.

Appendix 1 – Questionnaire in English

The questionnaire reported here is the literal translation of the original questionnaire which was in Dutch. The translation was completed by N.H.T and A.V.D.H

Question 1: Did you participate in a group session via Teams for informed consent?

- ☐ Yes
- ☐ No

If the answer above is “No” then this is the end of the questionnaire. If the answer above is “Yes’, then you can continue to question 2.

Question 2: Approximately how many participants attended your Teams session?

- ☐ Less than 5 participants
- ☐ Between 5 to 10 participants
- ☐ Between 11 to 15 participants
- ☐ More than 15 participants

Question 3: From which location did you join the digital informed consent session?

- ☐ Home
- ☐ Work
- ☐ Others

If the answer above is “Others”, please state which location you attended the session from.

Question 4: How do you rate the presence of other participants during this Teams session?

Numeric rating scale from 0 to 10. (0 is negative and 10 is positive)

Question 5: What are the advantages for you to have other participants in the Teams session? (e.g. it is good to hear questions from others).

Question 6: What are the disadvantages for you to have other participants in the Teams session? (e.g. I found it uncomfortable that other participants saw me participating in this study).

Question 7: Was it sufficiently emphasised that you could ask personal questions in a 1-to-1 conversation?

- ☐ Yes, I am aware of the option but did not need it
- ☐ Yes, I am aware of the option and have used it

- ☐ No, I am not aware of the option but did not need it
- ☐ No, I am not aware of the option and would have used it

Question 8: Healthy volunteers participate in the SWITCH ON study. Do you think this method of informed consent can also be used in studies involving people with a disease or condition and why?

- ☐ Yes
- ☐ No
- ☐ I would like to explain further

If the answer above is “I would like to explain further”, please elaborate your explanation.

Question 9: How do you feel about being allowed to log out only after you have signed the informed consent if you want to participate?

Numeric rating scale from 0 to 10. (0 is the negative and 10 is the positive)

Question 10: Have you ever had a face-to-face informed consent session in the past?

- ☐ Yes
- ☐ No

If the answer is “Yes”, please go to question 11 and 12. If the answer is “No”, please go to question 12.

Question 11: Which option do you prefer for an informed consent session?

- ☐ Face-to-face 1:1
- ☐ Face-to-face with 1 or 2 other participants
- ☐ Digital informed consent 1:1
- ☐ Digital informed consent with more than 2 participants

Question 12: Do you have any further comments regarding digital informed consent?
